# The vacuolar-type ATPase inhibitor archazolid increases tumor cell adhesion to endothelial cells by accumulating extracellular collagen

**DOI:** 10.1371/journal.pone.0203053

**Published:** 2018-09-11

**Authors:** Betty Luong, Rebecca Schwenk, Jacqueline Bräutigam, Rolf Müller, Dirk Menche, Iris Bischoff, Robert Fürst

**Affiliations:** 1 Institute of Pharmaceutical Biology, Goethe University Frankfurt, Frankfurt, Germany; 2 Department of Microbial Natural Products, Helmholtz-Institute for Pharmaceutical Research Saarland, Saarland University, Saarbrücken, Germany; 3 Kekulé-Institute for Organic Chemistry and Biochemistry, University of Bonn, Bonn, Germany; Medical College of Wisconsin, UNITED STATES

## Abstract

The vacuolar-type H^+^-ATPase (v-ATPase) is the major proton pump that acidifies intracellular compartments of eukaryotic cells. Since the inhibition of v-ATPase resulted in anti-tumor and anti-metastatic effects in different tumor models, this enzyme has emerged as promising strategy against cancer. Here, we used the well-established v-ATPase inhibitor archazolid, a natural product first isolated from the myxobacterium *Archangium gephyra*, to study the consequences of v-ATPase inhibition in endothelial cells (ECs), in particular on the interaction between ECs and cancer cells, which has been neglected so far. Human endothelial cells treated with archazolid showed an increased adhesion of tumor cells, whereas the transendothelial migration of tumor cells was reduced. The adhesion process was independent from the EC adhesion molecules ICAM-1, VCAM-1, E-selectin and N-cadherin. Instead, the adhesion was mediated by β1-integrins expressed on tumor cells, as blocking of the integrin β1 subunit reversed this process. Tumor cells preferentially adhered to the β1-integrin ligand collagen and archazolid led to an increase in the amount of collagen on the surface of ECs. The accumulation of collagen was accompanied by a strong decrease of the expression and activity of the protease cathepsin B. Overexpression of cathepsin B in ECs prevented the capability of archazolid to increase the adhesion of tumor cells onto ECs. Our study demonstrates that the inhibition of v-ATPase by archazolid induces a pro-adhesive phenotype in endothelial cells that promotes their interaction with cancer cells, whereas the transmigration of tumor cells was reduced. These findings further support archazolid as a promising anti-metastatic compound.

## Introduction

The vacuolar-type H^+^-ATPase (v-ATPase) is the major proton pump responsible for acidification of intracellular compartments in eukaryotic cells [1]. The enzyme consists of two multi-subunit complexes, the soluble V_1_ subcomplex catalyzing ATP hydrolysis and the transmembrane V_o_ subcomplex required for the proton transport across membranes [1,2]. In most cell types v-ATPases are only expressed in the endomembrane system to regulate and maintain the acidic pH of intracellular compartments such as lysosomes, endosomes, the Golgi apparatus, secretory granules and coated vesicles [3]. The function of v-ATPases is essential for cellular processes such as vesicular trafficking, receptor-mediated endocytosis and protein degradation and processing. In specialized cell types including osteoclasts and renal epithelial cells, v-ATPases can also be expressed on the plasma membrane, where they pump protons into the extracellular space [2–4]. In cancer cells v-ATPases are expressed on the plasma membrane in order to eliminate toxic cytosolic H^+^. Most importantly, v-ATPases contribute to the acidic tumor microenvironment, which leads to the activation of proteases, thus facilitating tumor cell migration, invasion and angiogenesis [5–7]. Since the inhibition of v-ATPase was shown to reduce the invasiveness of cancer cells and metastasis formation [8,9], this enzyme has emerged as a promising drug target in the recent years.

Archazolid A and B are highly potent and specific inhibitors of v-ATPases [10]. They were first isolated from the myxobacterium *Archangium gephyra* [11]. These compounds inhibit v-ATPase at low nanomolar concentrations [10,12] by binding to the subunit c of the V_o_ complex. As their biological activity is comparable to the v-ATPase inhibitors bafilomycin and concanamycin [10,11], archazolids are natural compounds of high interest that can be used both as a tool to study the consequences of v-ATPase inhibition and as a lead for drug development. Archazolids can be either produced by fermentation [11] or by total synthesis [13,14].

In the field of cancer research several studies reported on interesting pharmacological effects of archazolid: It reduced the migration of different invasive tumor cells in vitro and cancer cell metastasis in vivo in a breast tumor mouse model [15]. Furthermore, archazolid activated pathways of cellular stress response and apoptosis in highly invasive tumor cells [16]. In classically activated macrophages, archazolid selectively induced the generation of tumor necrosis factor α (TNFα), which may indirectly promote tumor suppression [17].

Up to now, the role of v-ATPases in endothelial cells has only rarely been investigated. The endothelium plays a crucial role in the pathogenesis and progression of cancer: The metastatic cascade includes local angiogenesis at the site of the primary tumor and adhesion of tumor cells at the site of metastasis [18]. Angiogenesis, the development of new blood vessels out of existing ones, depends on the proliferation, migration and differentiation of endothelial cells [19]. This process ensures the nutrient supply of the tumor and its growth [20]. Circulating cancer cells can adhere to the endothelium at distant sites. This adhesive interaction is mediated by receptors and corresponding ligands expressed on tumor and endothelial cells [18,21]. V-ATPases have been reported to regulate intracellular pH and cell migration in microvascular endothelial cells [22,23]. A recent study showed that the inhibition of v-ATPase by concanamycin prevented proliferation, reduced migration and impaired angiogenesis-related signaling in endothelial cells [24]. So far, there are no investigations on the role of endothelial v-ATPases for the process of tumor cell adhesion onto the endothelium. Thus, we were interested in the consequences of the inhibition of endothelial v-ATPase by archazolid on the interaction between endothelial and cancer cells. Various cell adhesion molecules on the endothelium, such as intercellular adhesion molecule 1 (ICAM-1), vascular cell adhesion protein (VCAM-1), E-selectin or N-cadherin [21] as well as integrins expressed on cancer cells have been reported to mediate cell adhesion of cancer cells onto endothelial cells [25–27]. Accordingly, we focused on these cell adhesion molecules and integrins. For the first time, our study revealed a link between the function of v-ATPases and the adhesion and transmigration properties of endothelial cells.

## Materials and methods

### Compounds

Archazolid A (hereinafter referred to as archazolid) was kindly provided by Prof. Dr. Rolf Müller (Saarland University) and Prof. Dr. Dirk Menche (University of Bonn). The compound was dissolved in dimethylsulfoxide (DMSO). Stocks of 10 mM archazolid were stored at -80°C and working stocks of 10 μM were stored at -20°C. For the treatment of cells archazolid was diluted in culture medium with a maximal concentration of 0.01% DMSO. Collagen G and fetal calf serum (FCS) were purchased from Biochrom (Berlin, Germany), recombinant tumor necrosis factor α (TNFα) from PeproTech (Hamburg, Germany), bovine serum albumin (BSA) from Carl Roth (Karlsruhe, Germany), CellTracker Green CMFDA Dye from Thermo Fisher Scientific (Schwerte, Germany), human plasma fibronectin (FC010) from Merck (Darmstadt, Germany) and laminin-411 from BioLamina (Sundbyberg, Sweden).

### Cell culture

Human umbilical vein endothelial cells (HUVECs) were obtained from PELOBiotech (Planegg/Martinsried, Germany) and immortalized human microvascular endothelial cells (HMEC-1) were provided by Centers for Disease Control and Prevention (lot 119223, CDC, Atlanta, GA, USA). Both types of endothelial cells were cultivated in collagen G-coated (10 μg/ml in phosphate-buffered saline [PBS]) flasks or seeded into collagen G-coated cell culture dishes or plates using endothelial cell growth medium (EASY ECGM, PELOBiotech) supplemented with 10% FCS, 100 U/ml penicillin, 100 μg/ml streptomycin (PAN-Biotech, Aidenbach, Germany), 2.5 μg/ml amphotericin B (PAN-Biotech) and a mixture of supplements provided by PELOBiotech. The human breast cancer cell line MDA-MB-231 (ACC-732) and the prostate cancer cell line PC-3 (ACC-465) were obtained from the Leibniz Institute DSMZ-German Collection of Microorganisms and Cell Cultures (Braunschweig, Germany). Both cell types were cultivated in Dulbecco’s modified eagle medium (DMEM) containing 4.5 g/l glucose (PAN-Biotech) supplemented with 10% FCS, 100 U/ml penicillin and 100 μg/ml streptomycin. All cell types were cultivated at 37°C and 5% CO_2_.

### Cytotoxicity assays

CellTiter-Blue Cell Viability Assay (Promega, Mannheim, Germany) was performed according to the manufacturer’s protocol for determining the cell viability of cells after treatment with archazolid. This assay is based on the ability of metabolically active cells to reduce resazurin which results in fluorescent resorufin. The CellTiter-Blue Reagent was added to the cells 4 h before the endpoint of treatment. Fluorescence was measured with an Infinite F200 pro microplate reader (Tecan, Männedorf, Switzerland) at 560 nm (excitation) and 590 nm (emission).

CytoTox 96 Non-Radioactive Cytotoxicity Assay (Promega) was performed according to the manufacturer’s instructions for determining the lactate dehydrogenase (LDH) release after treatment with archazolid. Lysis buffer was added to untreated cells 45 min before the end of treatment to induce the release of this enzyme. LDH is a cytosolic enzyme that is released by leaky cells. Released LDH catalyzes the enzymatic conversion of lactate to pyruvate which provides NADH for the conversion of iodonitrotetrazolium violet into a red formazan product in the presence of diaphorase. The absorbance was measured with a Varioskan Flash microplate reader (Thermo Fisher Scientific) at 490 nm.

### Lysotracker staining

LysoTracker Red DND-99 (Life Technologies, Thermo Fisher Scientific) is a dye to measure pH values in viable cells. HUVECs were cultured to confluence on collagen G-coated μ-slides (80826, ibidi, Martinsried, Germany) before they were treated with archazolid for 24 h. 1 μg/ml Hoechst 33342 (Sigma-Aldrich, Munich, Germany) was used to visualize the nuclei and 50 nM LysoTracker Red DND-99 was used to visualize the acidic compartments which correspond to the lysosomes. Both dyes were incubated for 10 min at 37°C before acquisition of single images by a Leica DMI6000 B fluorescence microscope (Leica Microsystems, Wetzlar, Germany).

### Cell adhesion assay

HUVECs were seeded in collagen G-coated 24-well plates and grown to confluence for two days before treatment. The cells were incubated with indicated concentrations of archazolid for 24 h. Untreated MDA-MB-231 or PC-3 cells were labeled with CellTracker Green CMFDA Dye (5 μM in serum-free DMEM, 37°C) for 30 min before 100,000 cells per well were added to HUVECs and were allowed to adhere for various time points at 37°C. Non-adherent tumor cells were washed off three times with PBS containing Ca^2+^ and Mg^2+^. Tumor cell adhesion was determined by fluorescence measurements with an Infinite F200 pro microplate reader (Tecan) at 485 nm (excitation) and 535 nm (emission).

For blocking the integrin β1 subunit on MDA-MB-231 or PC-3 cells, CellTracker Green-labeled MDA-MB-231 or PC-3 cells were incubated with an anti-integrin β1 antibody (P5D2, ab24693, Abcam, Cambridge, United Kingdom) at a concentration of 1 μg antibody per one million cells in 1 ml DMEM. Before adding to archazolid-treated HUVECs, MDA-MB-231 or PC-3 cells were washed once with DMEM. For blocking the integrin β1 subunit on HUVECs, the cells were incubated with the anti-integrin β1 antibody (0.1 μg/well in ECGM). HUVECs were washed once with ECGM before untreated MDA-MB-231 or PC-3 cells were added to HUVECs.

For the adhesion of MDA-MB-231 or PC-3 cells onto extracellular matrix (ECM) components 24-well plates were coated with collagen G (10 μg/ml in PBS), human plasma fibronectin (10 μg/ml PBS) or laminin-411 (10 μg/ml in Dulbecco’s PBS [DPBS] containing Ca^2+^ and Mg^2+^) at 4°C overnight. The adhesion of MDA-MB-231 and PC-3 cells onto these three most prominent ECM components was carried out as described above (10 min adhesion at 37°C).

### Endothelial transmigration assay

HUVECs were grown on a porous filter membrane (Transwell insert, polycarbonate membrane, 8 μm pores; Corning, New York, USA) for 48 h and were treated as indicated. Untreated MDA-MB-231 cells were labeled with CellTracker Green CMFDA Dye (as described in the section cell adhesion assay) and resuspended in medium 199 (PAN-Biotech) containing 0.1% BSA. HUVECs were washed twice with medium 199 containing 0.1% BSA before MDA-MB-231 cells were allowed to transmigrate through the endothelial monolayer for 24 h. Medium 199 containing 0.1% BSA was used as negative control and medium 199 containing 20% FCS was used as chemoattractant for transmigration in the lower compartment. Non-migrated cells remaining in the upper compartment were carefully removed using a cotton swab. Transmigrated cells were lysed in radioimmunoprecipitation assay (RIPA) buffer and transmigration was quantified by measuring the fluorescence signal at 485 nm (excitation) and 535 nm (emission).

### Quantitative polymerase chain reaction (qPCR)

HUVECs were grown to confluence on 6-well plates before they were treated with archazolid for 12 h. The cells were induced to upregulate the gene expression of cell adhesion molecules by TNFα. RNA was isolated using the RNeasy Mini Kit from Qiagen (Hilden, Germany) according to the manufacturer’s protocol. On-column DNase digestion was performed to remove genomic DNA. RNA was transcribed into cDNA by Superscript II (Life Technologies, Thermo Fisher Scientific). qPCR experiments were performed using a StepOnePlus System (Applied Biosystems, Thermo Fisher Scientific) and data was analyzed by the StepOne and StepOnePlus Software v2.3. Power SYBR Green PCR Master Mix (Life Technologies) and the comparative C_T_ quantitation method (2^-ΔΔCT^) were used. The following primers were used for qPCR experiments: ICAM-1 (forward 5’-CTG CTC GGG GCT CTG TTC-3’; reverse 5’-AAC AAC TTG GGC TGG TCA CA-3’), VCAM-1 (forward 5’-CCA CAG TAA GGC AGG CTG TAA-3’; reverse 5’-GCT GGA ACA GGT CAT GGT CA-3’), E-selectin (forward 5’-AGA TGA GGA CTG CGT GGA GA-3’; reverse 5’-GTG GCC ACT GCA GGA TGT AT-3’), N-cadherin (forward 5’-CAG GAA AAG TGG CAA GTG GC-3’; reverse 5’-AGG AAA AGG TCC CCT GGA GT-3’) and GAPDH (forward 5’-CCA CAT CGC TCA GAC ACC AT-3’; reverse 5’-TGA AGG GGT CAT TGA TGG CAA-3’). The threshold cycle of the target gene was normalized to the threshold cycle of the *GAPDH* gene.

### Flow cytometry

HUVECs were grown to confluence on 12-well plates before they were treated with archazolid for 24 h. Cells were treated with TNFα for 24 h to induce the expression of cell adhesion molecules. Subsequently, the cells were detached with HyClone HyQTase (GE Healthcare, Freiburg, Germany). In the case of ICAM-1 the detached cells were fixed with 4% formaldehyde (Polysciences, Hirschberg an der Bergstraße, Germany) in PBS for 10 min and washed once with PBS before incubating with the fluorescein isothiocyanate (FITC)-labeled anti-human CD54 (mouse, ICAM-1) antibody (MCA1615F, Biozol, Eching, Germany) at room temperature for 45 min. For all other proteins, the cells were not fixed and washed once with PBS before incubating with the antibodies phycoerythrin (PE)-labeled anti-human CD106 (mouse, VCAM-1), PE-labeled anti-human CD62E (mouse, E-selectin) and PE-labeled anti-human CD325 (mouse, N-cadherin) from Becton Dickinson on ice for 45 min. These antibodies were diluted in PBS containing 0.2% BSA. The surface expression of cell adhesion molecules was measured by flow cytometry (FACSVerse, Becton Dickinson, Heidelberg, Germany).

### Immunofluorescence staining of viable cells

To stain the surface collagen on HUVECs, cells were incubated with an anti-human collagen antibody (rabbit, 1:40, ab36064, Abcam) on ice for 30 min. The staining procedure was performed on ice to ensure that surface proteins or antibodies are not endocytosed. The cells were washed once with PBS containing Ca^2+^ and Mg^2+^ before they were fixed with Roti-Histofix (Carl Roth). Alexa Fluor 488-conjugated anti-rabbit antibody (goat, 1:400, A11008, Life Technologies) was used as secondary antibody and Hoechst 33342 (1 μg/ml, Sigma-Aldrich) was used to visualize nuclei.

### Western blot

Confluent HUVECs in 6-well plates were treated as indicated. Cells were washed with ice-cold PBS and lysed with RIPA buffer supplemented with protease inhibitors (Complete Mini EDTA-free; Roche, Mannheim, Germany), sodium orthovanadate, sodium fluoride, phenylmethylsulphonyl fluoride, β-glycerophosphate, sodium pyrophosphate and H_2_O_2_. Protein determination was performed using the Pierce BCA Protein Assay Kit (Thermo Fisher Scientific). Equal amounts of proteins (10–20 μg) were separated by sodium dodecyl sulfate-polyacrylamide gel electrophoresis (SDS-PAGE; Bio-Rad Laboratories, Munich, Germany). Separated proteins were transferred onto polyvinylidene difluoride membranes by tank blotting (Bio-Rad Laboratories) for immunodetection. Membranes were blocked with 5% bolting-grade milk powder (Carl Roth) in TBS containing 0.1% Tween 20 (Sigma-Aldrich). The following antibodies were used: mouse anti-human cathepsin B antibody (IM27L, Merck) (1:500), mouse anti-β-actin-peroxidase antibody (A3854, Sigma-Aldrich) (1:100,000) and anti-mouse IgG horse radish peroxidase (HRP)-linked antibody (7076, Cell Signaling, Frankfurt, Germany) (1:5,000). ImageJ version 1.49m was used for densitometric analysis.

### Cathepsin B activity assay

Cathepsin B activity assay was performed as described in the publication by Kubisch *et al*. [28]. Confluent HUVECs or HMEC-1 seeded in 6-well plates were treated as indicated. Cells were washed with PBS and lysed with the non-denaturating M-PER mammalian protein extraction reagent (78501, Thermo Fisher Scientific) supplemented with protease inhibitors (Complete Mini EDTA-free, Roche), sodium orthovanadate, sodium fluoride, phenylmethylsulphonyl fluoride. The fluorogenic cathepsin B substrate Z-Arg-Arg-7-amido-4-methylcoumarin hydrochloride (C5429, Sigma-Aldrich) was added to 30 μg of the cell lysate diluted in assay buffer supplemented with 2 mM L-cysteine (C7880, Sigma-Aldrich) and incubated for 30 min at 40°C. Fluorescence was measured at 348 nm (excitation) and 440 nm (emission) with a microplate reader (Varioskan Flash, Thermo Fisher Scientific). The intensity of the fluorescence signal corresponded to the cathepsin B enzyme activity. For background subtraction the cathepsin B inhibitor CA-074Me (Enzo Life Sciences, Lörrach, Germany) was added to an additional reaction.

### Transfection of HUVECs

The HUVEC Nucleofector Kit (Lonza, Cologne, Germany) was used to transfect HUVECs. The transfection was performed according to the manufacturer’s protocol using 2.5 μg plasmid DNA for 500,000 cells (Nucleofector 2b Device, Lonza). 48 h after transfection the cells were treated for further experiments. The addgene plasmid #11249 hCathepsin B was kindly provided by Hyeryun Choe [29]. hCathepsin B was digested with PmeI and XbaI and the linear DNA fragment not corresponding to the human *CTSB* gene was religated to generate the empty pcDNA3.1 (-) delta MCS plasmid that was used for control transfections. The original backbone of hCathepsin B is the pcDNA3.1 (-) from Thermo Fisher Scientific. The control vector pcDNA3.1 (-) delta MCS used for our transfections was cloned on the basis of hCathepsin B and is therefore lacking almost the whole part of the multiple cloning site of the pcDNA3.1 (-).

### Statistical analysis

Statistical analyses were performed using GraphPad Prism 5.0 (San Diego, USA). One-way ANOVA followed by Tukey’s post-hoc test or unpaired t-test was used for the evaluation of a minimum of three independent experiments. The numbers of independently performed experiments (n) are stated in the corresponding figure legends. p ≤ 0.05 was considered as statistically significant. Data are expressed as mean ± standard error of the mean (SEM).

## Results

### Archazolid is functionally active but not cytotoxic to HUVECs

**Fig 1:**
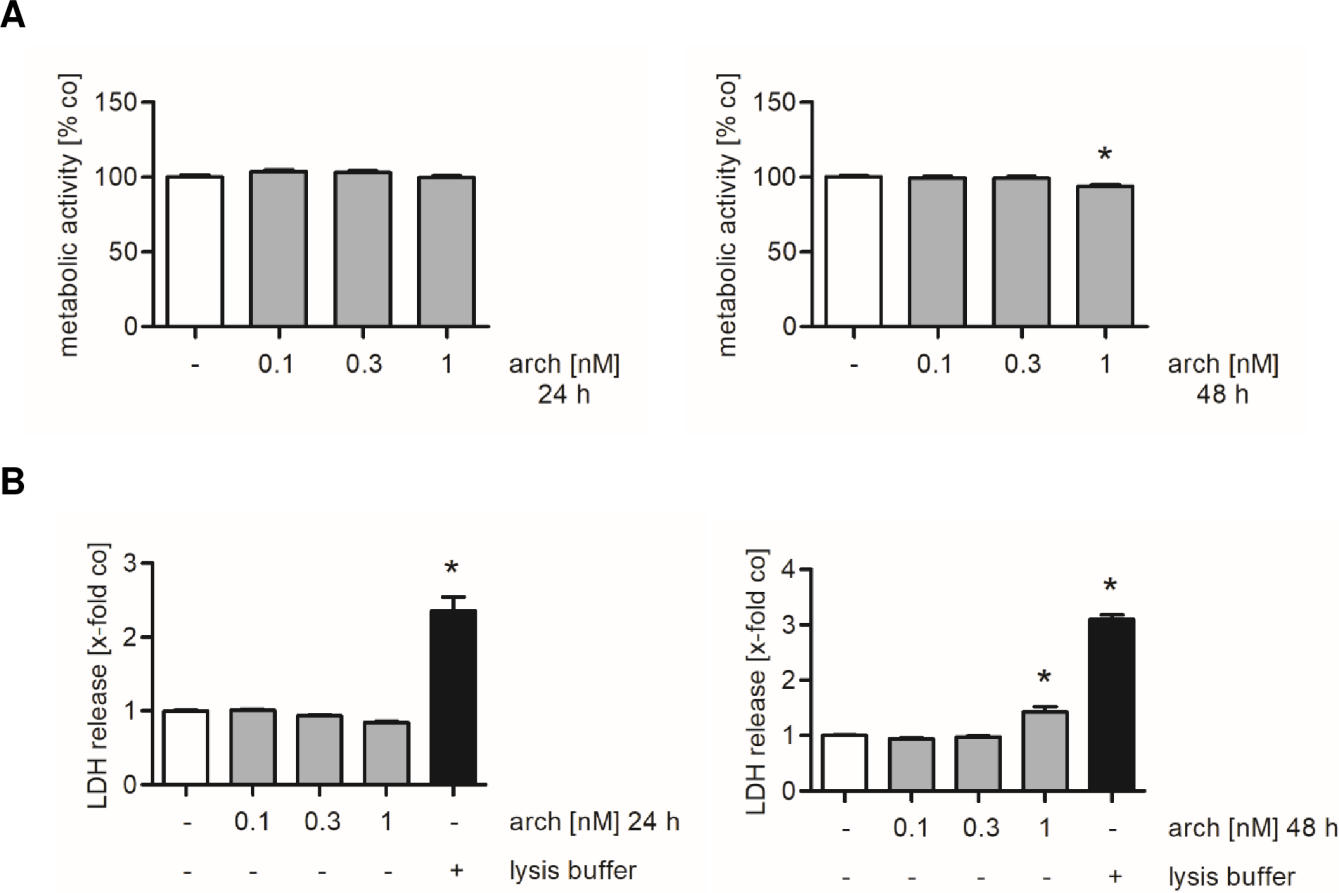
Treatment of HUVECs with archazolid for 24 h does not evoke cytotoxic effects. Confluent HUVECs were treated either with archazolid (arch) or DMSO (co) for 24 h and 48 h. Both the metabolic activity (A) and the release of LDH (B) were determined. Treatment with lysis buffer served as positive control for LDH release. (A, B) Data are expressed as mean ± SEM (n = 3). *p ≤ 0.05 versus DMSO control.

Since the v-ATPase inhibitor archazolid has never been used for studies in endothelial cells, we first performed cytotoxicity assays. We treated confluent HUVECs with up to 1 nM archazolid for 24 and 48 h and observed that this treatment has neither an influence on the metabolic activity nor on the release of LDH after 24 h (Fig 1A and 1B, left panels). The metabolic activity and the release of LDH were only slightly affected by the highest concentration of archazolid after 48 h (Fig 1A and 1B, right panels). Consequently, the following experiments were all carried out after 24 h (or less) of archazolid treatment in order to exclude any cytotoxic effects of archazolid within our experimental settings.

**Fig 2:**
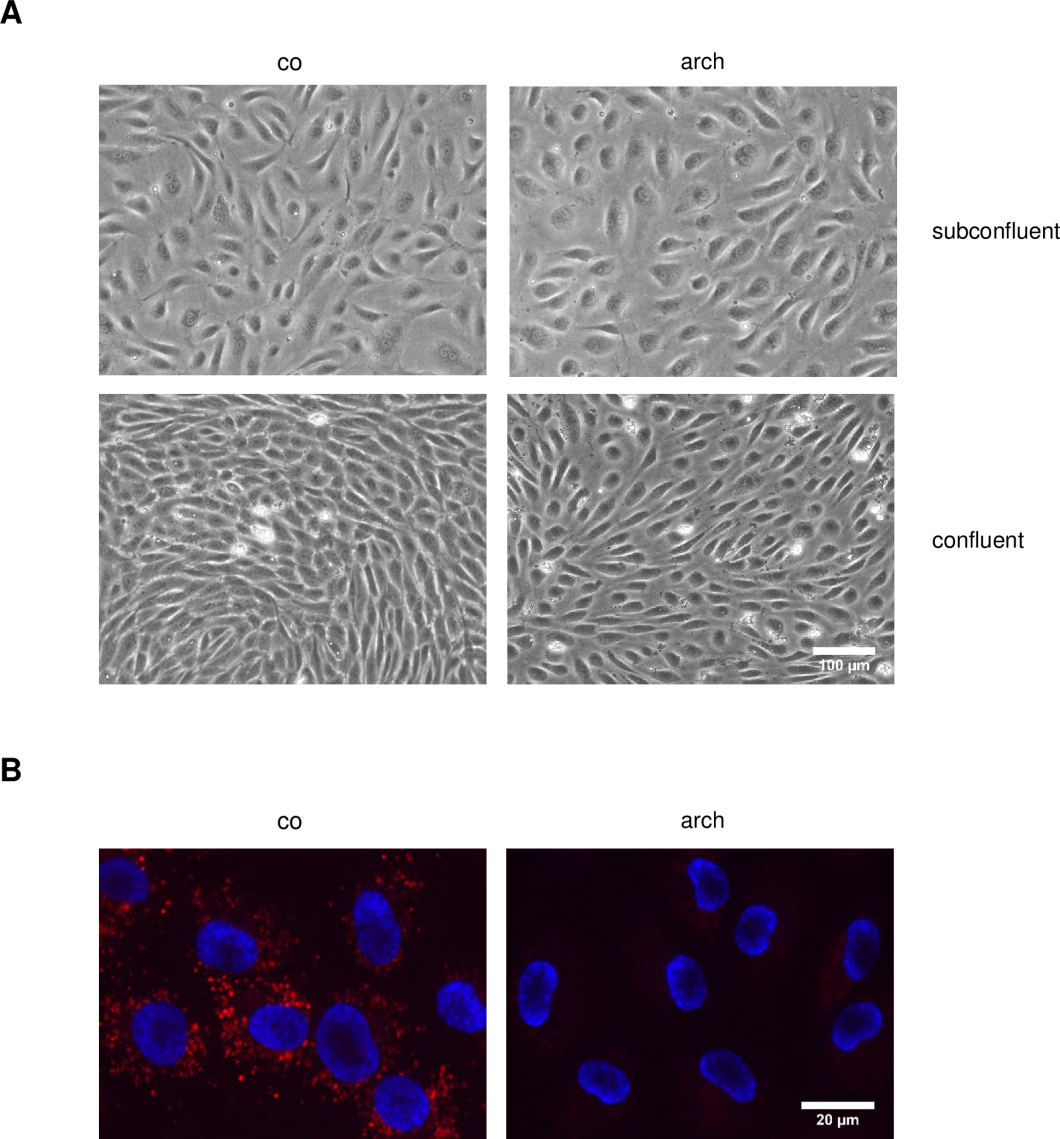
Archazolid alter the cell morphology and is functionally active in HUVECs. Confluent HUVECs were treated with 1 nM archazolid (arch) or DMSO (co). (A) Microscopic images of fixed cells were taken. Scale bar represents 100 μm. (B) Fluorescence microscopic images of viable cells were taken. LysoTracker Red DND-99 was used to visualize the acidic compartments (lysosomes) and Hoechst 33342 (blue) staining was used to visualize nuclei. Scale bar represents 20 μm. (A, B) One representative image out of three independently performed experiments is shown.

Microscopic analysis revealed that also the integrity of the endothelial monolayer was not affected by archazolid, but the cells showed a slightly different morphology (Fig 2A): Archazolid-treated cells were swollen compared to control cells, which was not unexpected, as vacuolation of the endoplasmic reticulum (ER) has been described for other cell types and is typical for v-ATPase inhibitors [11,16,24,30]. This effect was obvious both in subconfluent and in confluent cells (Fig 2A).

Inhibition of v-ATPase prevents the acidification of lysosomes [1,31]. Using the cell-permeable dye LysoTracker Red DND-99, it is possible to label the acidic lysosomes in living cells. Thus, this dye can serve as an indicator of v-ATPase inhibition. To proof that archazolid is also functionally active as a v-ATPase inhibitor in HUVECs, cells were treated with 1 nM archazolid before they were incubated with LysoTracker Red DND-99 and Hoechst 33342. As shown in Fig 2B, the red vesicular staining corresponding to acidified lysosomes in control cells disappeared completely after treatment with archazolid. In summary, archazolid treatment for 24 h was not cytotoxic to quiescent HUVECs, but inhibited the functionality of the v-ATPase.

### Archazolid increases the adhesion of tumor cells onto endothelial cells but decreases the transendothelial migration of tumor cells through the endothelial monolayer

**Fig 3:**
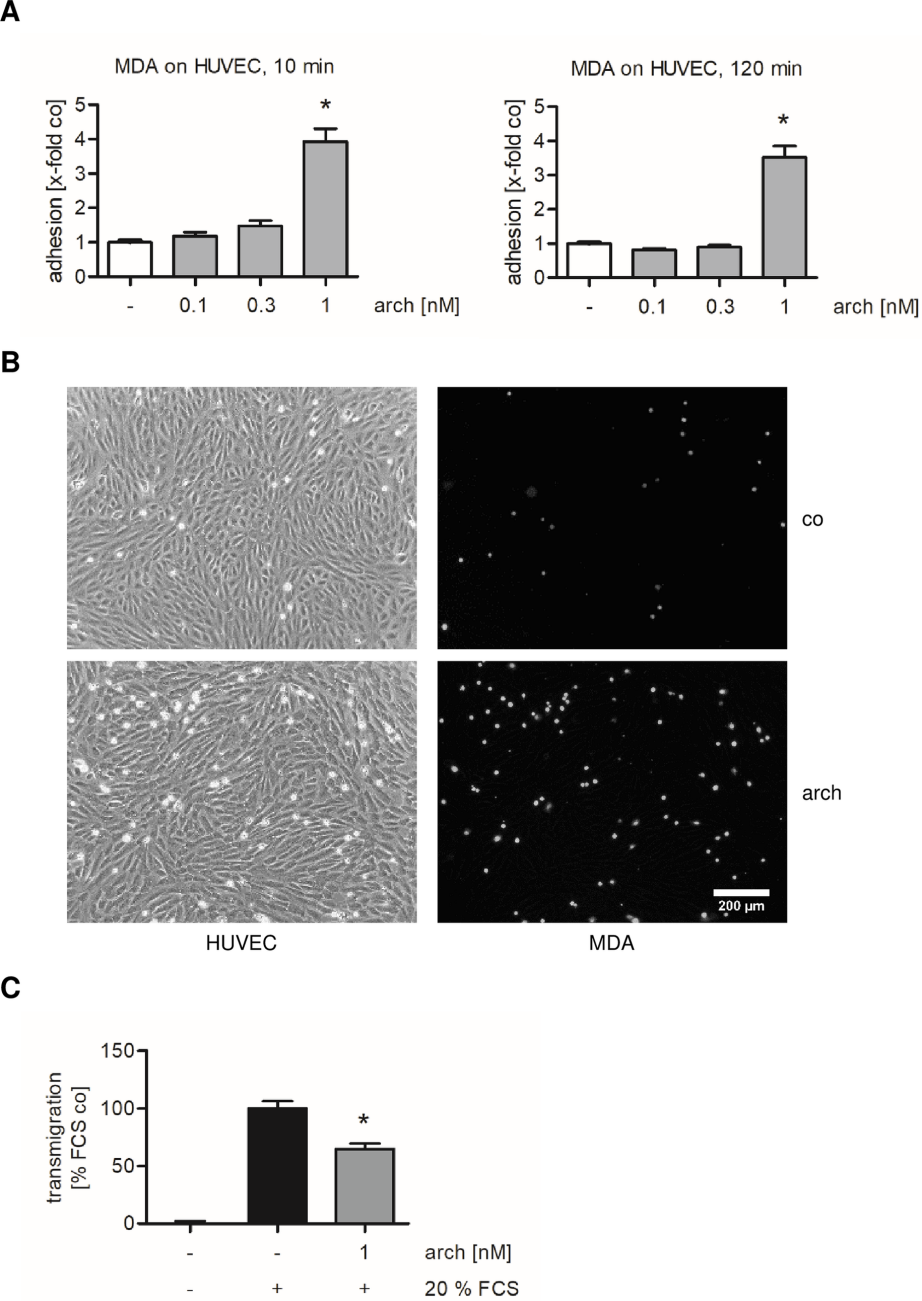
V-ATPase inhibition by archazolid increases the adhesion of MDA-MB-231 cells onto HUVECs and decreases their transendothelial migration. (A, B) Confluent HUVECs were treated with archazolid (arch) or DMSO (co) for 24 h. Untreated MDA-MB-231 cells were stained with CellTracker Green CMFDA Dye and were added to the HUVEC monolayer. The cells were allowed to adhere for 10 min and 120 min. Non-adherent MDA-MB-231 cells were washed off. (A) The adhesion was quantified by measuring the fluorescence signal using a plate reader. Data are expressed as mean ± SEM (n = 7). *p ≤ 0.05 versus DMSO control. (B) Microscopic images show unlabeled HUVECs and CellTracker Green CMFDA Dye-labeled MDA-MB-231 cells after 10 min of adhesion. Scale bar represents 200 μm. One representative image out of three independently performed experiments is shown. (C) HUVECs were grown on a porous filter membrane and were treated with archazolid for 24 h. Untreated CellTracker Green-labeled MDA-MB-231 cells were allowed to transmigrate through the endothelial monolayer for 24 h. Transmigrated cells were quantified by measuring the fluorescence signal. Data are expressed as mean ± SEM (n = 5). *p ≤ 0.05 versus FCS control.

We analyzed the adhesion of MDA-MB-231 cells onto HUVECs. Confluent HUVECs were treated with up to 1 nM archazolid for 24 h. Untreated MDA-MB-231 cells were labeled with CellTracker Green CMFDA Dye. Interestingly, v-ATPase inhibition strongly increased the attachment of the metastatic breast carcinoma cell line MDA-MB-231 onto HUVECs after 10 and 120 min of adhesion (Fig 3A and 3B). We also investigated the influence of archazolid on the transendothelial migration of MDA-MB-231 cells. HUVECs seeded in a Boyden chamber were treated with 1 nM archazolid for 24 h. CellTracker Green-labeled MDA-MB-231 cells (not treated with archazolid) were allowed to transmigrate through the endothelial monolayer for 24 h. As shown in Fig 3C, archazolid significantly decreased the transendothelial migration of MDA-MB-231 cells.

**Fig 4:**
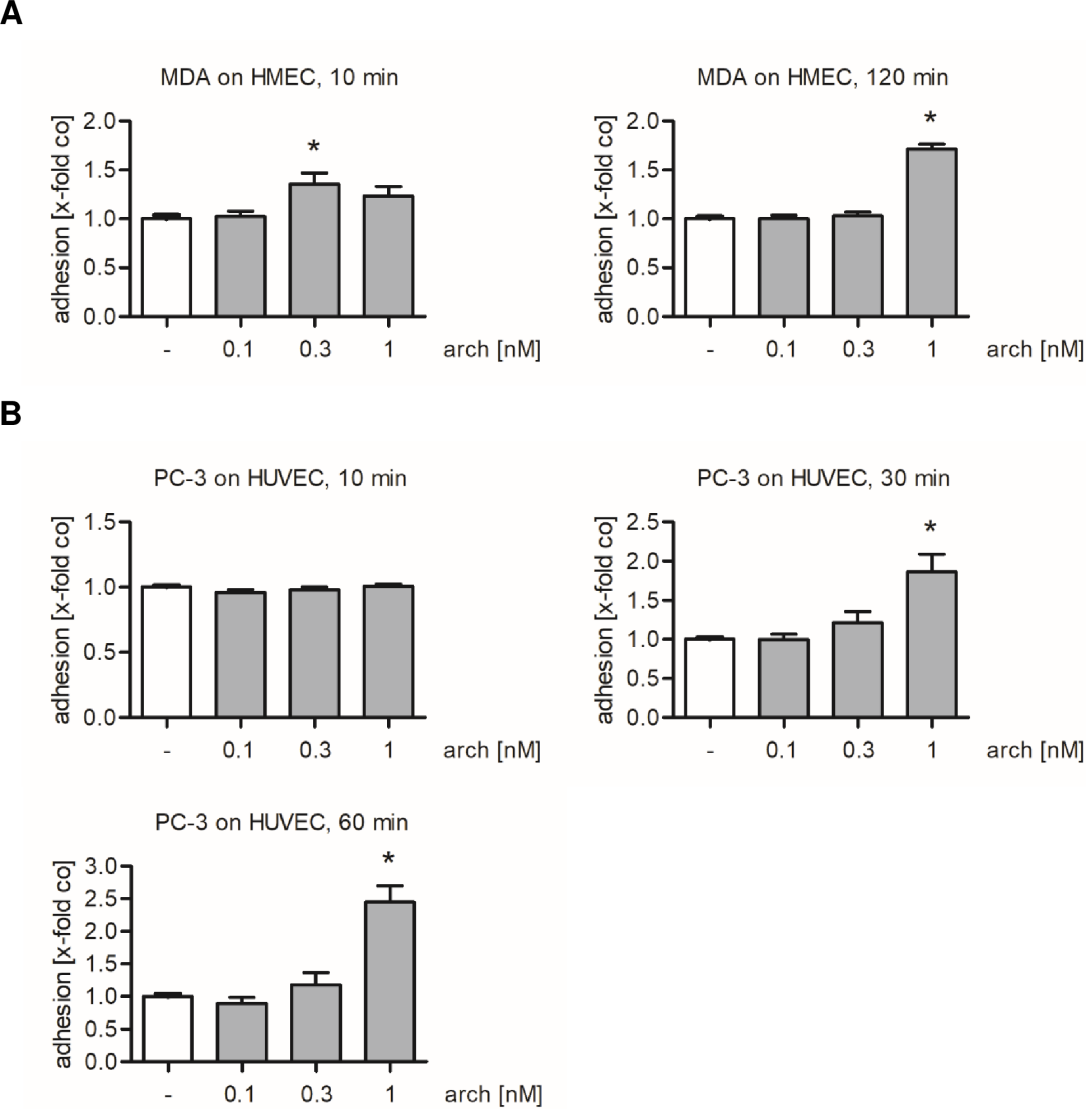
V-ATPase inhibition by archazolid increases the adhesion of MDA-MB-231 cells onto HMEC-1 and the adhesion of PC-3 cells onto HUVECs. Confluent endothelial cells were treated with archazolid (arch) or DMSO (co) for 24 h. (A) Untreated MDA-MB-231 cells were stained with CellTracker Green CMFDA Dye and added to an HMEC-1 monolayer. The cells were allowed to adhere for 10 and 120 min. Non-adherent MDA-MB-231 cells were washed off. (B) Untreated PC-3 cells were stained with CellTracker Green CMFDA Dye and added to the HUVEC monolayer. The cells were allowed to adhere for 10, 30 and 60 min. Non-adherent PC-3 cells were washed off. (A, B) The adhesion was quantified by measuring the fluorescence signal using a plate reader. Data are expressed as mean ± SEM (n = 3). *p ≤ 0.05 versus DMSO control.

The influence of archazolid on tumor cell adhesion was not only studied in HUVECs, which represent macrovascular endothelial cells, but also in microvascular HMEC-1 cells. Moreover, besides the breast cancer cell line MDA-MB-231, also PC-3 prostate cancer cells were used as a second metastatic cancer cell line. Archazolid treatment of endothelial cells increased the attachment of MDA-MB-231 cells onto the HMEC-1 monolayer after 120 min of adhesion (Fig 4A) and increased the attachment of PC-3 cells onto the HUVEC monolayer after 30 and 60 min of adhesion (Fig 4B). Of note, the adhesion of non-metastatic Jurkat cells, an acute T cell leukemia cell line, remained unaffected after treatment of HUVECs with archazolid (S1A Fig). Taken together, archazolid treatment augmented the adhesive properties of both micro- and macrovascular endothelial cells for metastatic tumor cells, but not for non-metastatic ones. Of note, cancer cell adhesion onto archazolid-activated endothelial cells increased with the time of adhesion.

### The archazolid-induced increase in tumor cell adhesion onto HUVECs is independent from endothelial cell adhesion molecules

**Fig 5:**
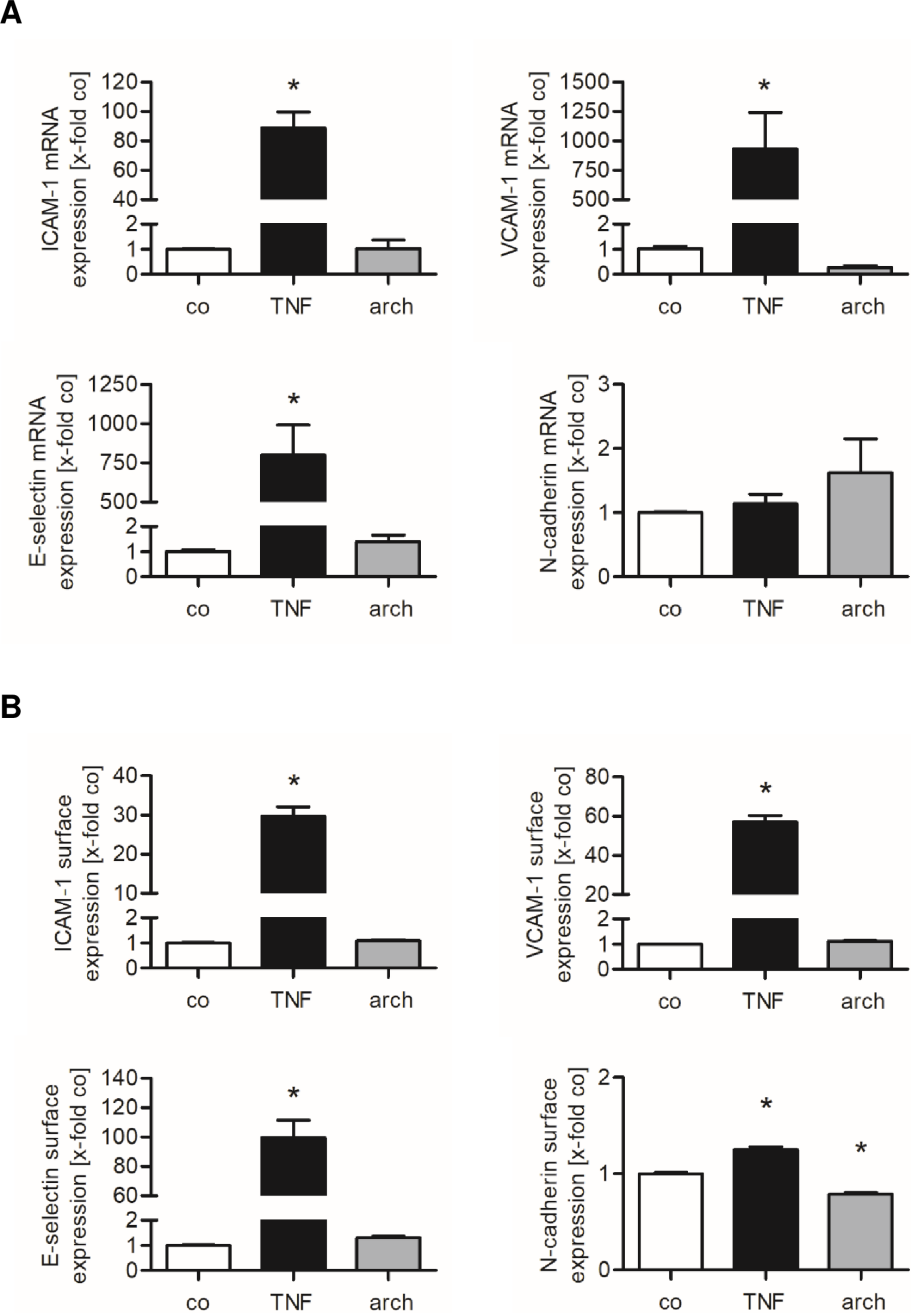
Archazolid does not upregulate the expression of cell adhesion molecules in HUVECs. (A) Confluent HUVECs were treated with 1 nM archazolid (arch) or DMSO (co) for 12 h. The expression of ICAM-1, VCAM-1, E-selectin and N-cadherin was analyzed on the mRNA level by qPCR experiments. (B) Confluent HUVECs were treated with 1 nM archazolid (arch) or DMSO (co) for 24 h. The surface expression of ICAM-1, VCAM-1, E-selectin and N-cadherin was analyzed by flow cytometry. (A, B) Data are expressed as mean ± SEM (n = 3). *p ≤ 0.05 versus DMSO control. TNFα (TNF) served as positive control to induce both the mRNA (1 ng/ml TNF) and the cell surface expression (10 ng/ml TNF) of ICAM-1, VCAM-1, E-selectin and N-cadherin.

The adhesion of tumor cells onto the endothelium is in principle similar to that of leukocytes, but slightly differs in the molecules that mediate the adhesion process. Ligands for the endothelial cell adhesion molecules ICAM-1, VCAM-1, E-selectin and N-cadherin were found to be expressed on tumor cells and to mediate tumor-endothelial cell interaction [21]. Inhibition of the v-ATPase might affect the expression of endothelial cell adhesion molecules on mRNA or protein levels. To determine the mRNA expression of ICAM-1, VCAM-1, E-selectin and N-cadherin, HUVECs were treated with archazolid for 12 h. TNFα is known to upregulate the expression of ICAM-1, VCAM-1 and E-selectin [32] and, thus, served as positive control. Quantitative real-time PCR showed that v-ATPase inhibition in HUVECs did not alter the mRNA levels of ICAM-1, VCAM-1, E-selectin and N-cadherin (Fig 5A). The protein expression of these adhesion molecules on the surface of endothelial cells was analyzed by flow cytometry. Archazolid (1 nM, 24 h) did not affect the cell surface expression of ICAM-1, VCAM-1, E-selectin and N-cadherin (Fig 5B).

### β1-integrins on tumor cells mediate the archazolid-induced cell adhesion

**Fig 6:**
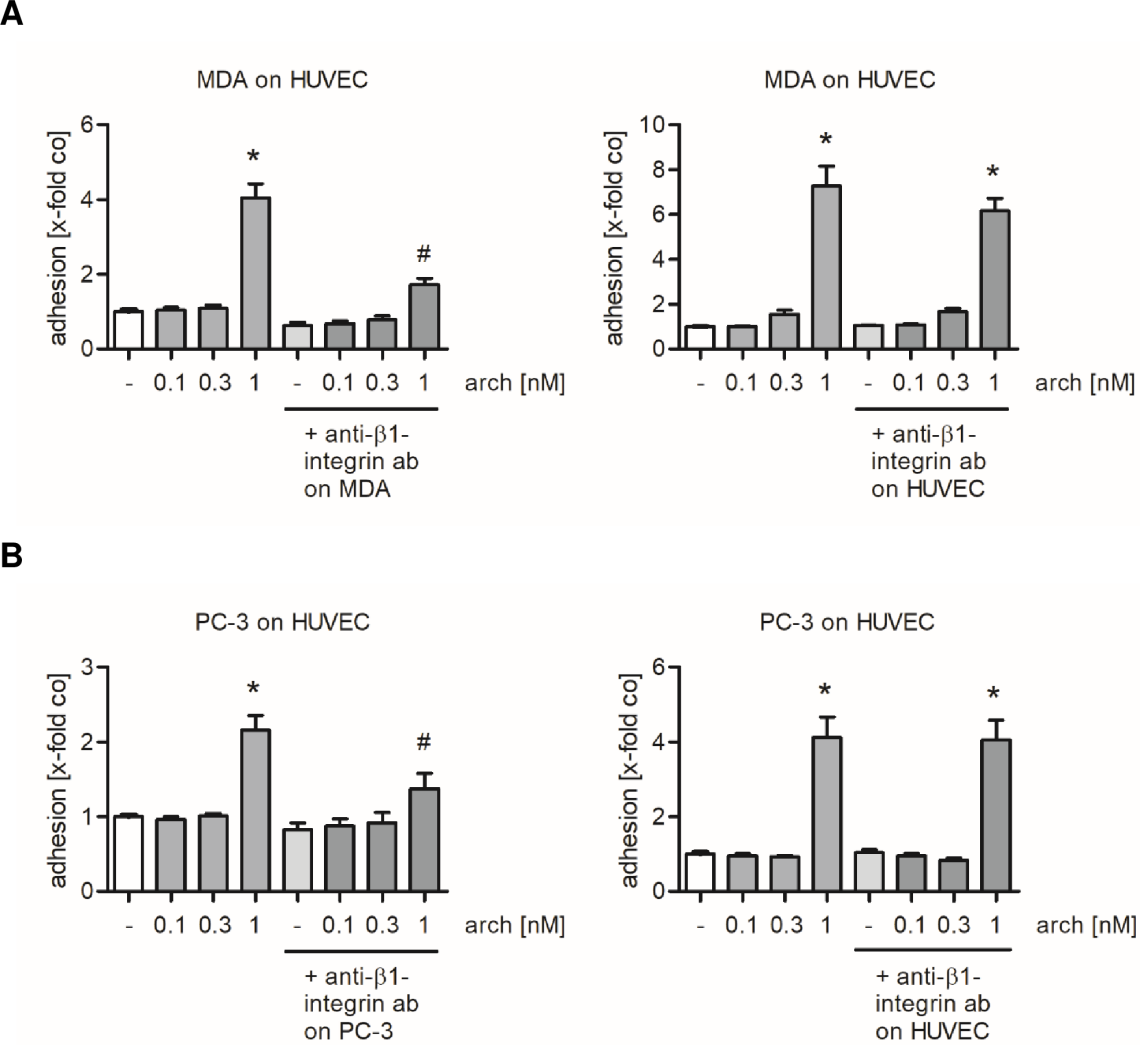
Blocking integrin β1 subunits on MDA-MB-231 or PC-3 cells prevents the archazolid-mediated increase in tumor cell adhesion. Confluent HUVECs were treated either with archazolid or DMSO for 24 h. MDA-MB-231 or PC-3 cells were stained with CellTracker Green CMFDA Dye. (A) The cell adhesion assay was performed after the integrin β1 subunit was blocked by a monoclonal antibody for 30 min on either MDA-MB-231 (n = 6) cells or HUVECs (n = 3). MDA-MB-231 cells were allowed to adhere for 10 min. (B) The cell adhesion assay was performed after the integrin β1 subunit was blocked by a monoclonal antibody for 30 min on either PC-3 cells (n = 5) or HUVECs (n = 3). PC-3 cells were allowed to adhere for 60 min. (A, B) Non-adherent tumor cells were washed off. The adhesion was quantified by measuring the fluorescence signal using a plate reader. Data are expressed as mean ± SEM. *p ≤ 0.05 versus DMSO control, ^#^p ≤ 0.05 versus 1 nM archazolid.

Besides ICAM-1, VCAM-1, E-selectin and N-cadherin, also integrins are able to mediate the process of cell adhesion [33–35]. Since none of the cell adhesion molecules expressed on HUVECs were regulated upon archazolid treatment, we considered integrins as potential interaction partners. Within this protein family β1-integrins have been reported to mediate tumor cell adhesion onto quiescent endothelial cells [25]. In order to elucidate the role of β1-integrins for the archazolid-induced tumor cell adhesion, the integrin β1-subunit was blocked either on MDA-MB-231 cells, PC-3 cells or on HUVECs. (Of note, as in all experiments throughout this study, only endothelial cells were treated with archazolid.) After blocking β1-integrins on MDA-MB-231 or PC-3 cells, the archazolid-induced tumor cell adhesion was reduced almost to control level (Fig 6A and 6B, left panels), whereas blocking of β1-integrins on HUVECs had no significant effect on the increase of tumor cell adhesion by v-ATPase inhibition (Fig 6A and 6B, right panels).

### MDA-MB-231 and PC-3 cells preferentially adhere to the β1-integrin ligand collagen

**Fig 7:**
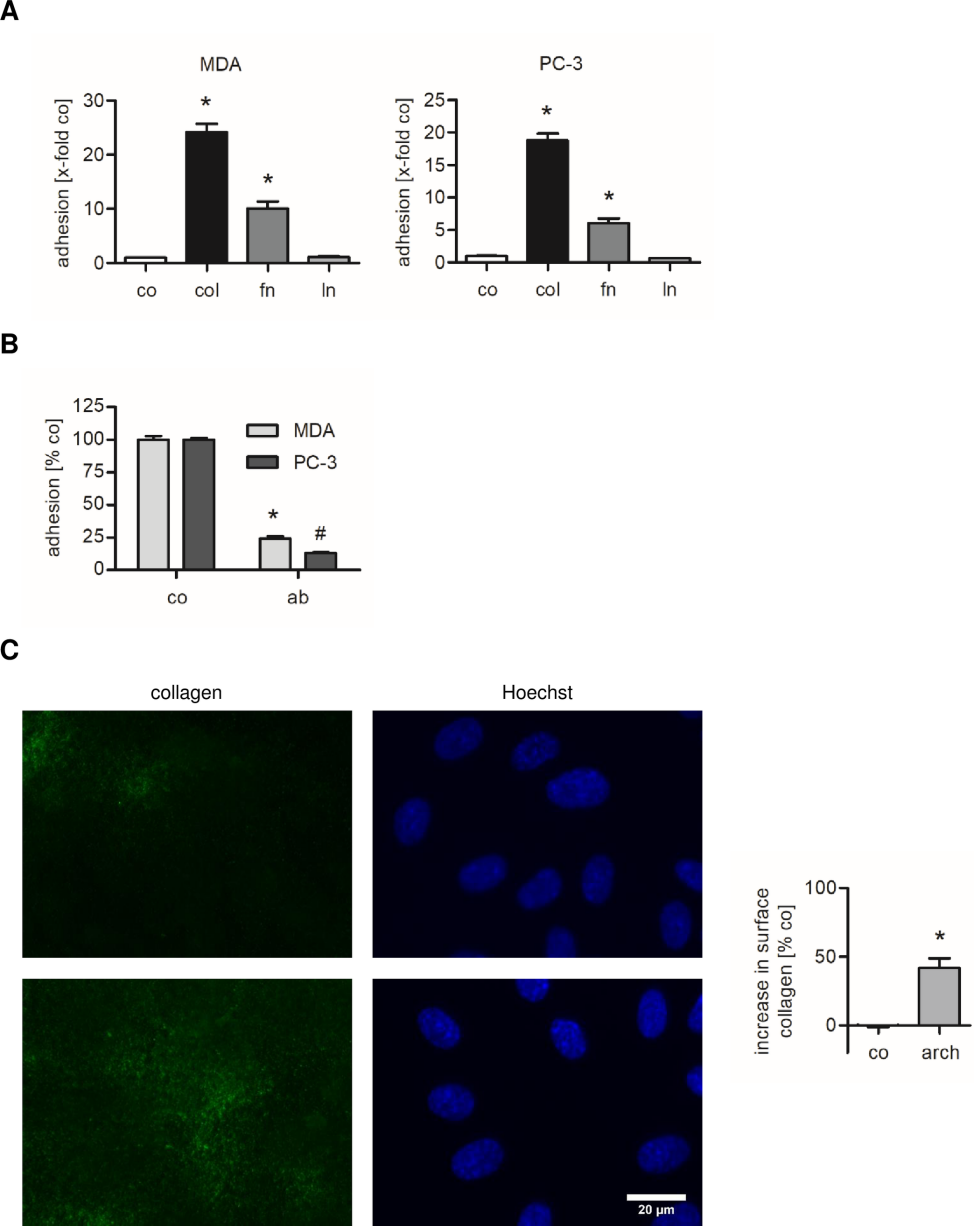
Collagen is the major ECM component mediating MDA-MB-231 and PC-3 cell adhesion. **Archazolid increases the amount of extracellular collagen on HUVECs.** (A) 24-well plates were coated with 10 μg/ml collagen (col), fibronectin (fn) or laminin (ln) or were left uncoated (co). Untreated MDA-MB-231 or PC-3 cells were stained with CellTracker Green CMFDA Dye and added into ECM-coated or uncoated wells. After 10 min of incubation, non-adherent cells were washed off. Adhesion was quantified by measuring the fluorescence signal using a plate reader. Data are expressed as mean ± SEM (n = 3). *p ≤ 0.05 versus uncoated control. (B) A cell adhesion assay onto collagen was performed with untreated MDA-MB-231 or PC-3 cells (co) and MDA-MB-231 or PC-3 cells on which the integrin β1 subunit was blocked by a monoclonal antibody (ab). Data are expressed as mean ± SEM (n = 3). *^,#^p ≤ 0.05 versus control. (C) Confluent HUVECs were treated with 1 nM archazolid (arch) or DMSO (co) for 24 h. Surface collagen (green) was detected by immunofluorescence staining of viable cells and nuclei (blue) were visualized by Hoechst 33342 staining. Scale bar represents 20 μm. One representative image out of three independently performed experiments is shown. The increase in extracellular collagen was quantified. Data are expressed as mean ± SEM (n = 3). *p ≤ 0.05 versus DMSO control.

Depending on their α subunit, β1-integrins have a variety of ligands including extracellular matrix (ECM) components such as collagen, fibronectin and laminin [35]. Therefore, we hypothesized that archazolid treatment of endothelial cells might lead to an upregulation of these components. MDA-MB-231 and PC-3 cells were allowed to adhere onto plastic that was coated with these ECM components. This cell adhesion assay revealed that MDA-MB-231 as well as PC-3 cells favor the interaction with the ECM component collagen, as the adhesion onto collagen is much higher than onto the uncoated plastic control (Fig 7A). MDA-MB-231 and PC-3 cells also adhered to fibronectin-coated plastic, but to a much lesser extent compared to the collagen coating. Therefore, we focused on the interaction between these two tumor cell lines and collagen. Blocking of the integrin β1 subunit on MDA-MB-231 and PC-3 cells clearly abolished the interaction with collagen (Fig 7B), indicating that the attachment of these tumor cells to collagen is mediated by β1-integrins.

### Archazolid leads to an accumulation of collagen on the endothelial cell surface

Since collagen is the major ECM component MDA-MB-231 and PC-3 cells interact with, the next step was to prove whether v-ATPase inhibition influences the amount of collagen expressed by HUVECs as extracellular matrix. To detect collagen on the endothelial surface, archazolid-treated HUVECs were labeled with an antibody against collagen type I-IV on ice to prevent endocytosis and to ensure that the antibody does not bind to intracellular collagen. Interestingly, archazolid increased the amount of surface collagen on HUVECs by about 50% (Fig 7C). Control stainings were performed using an antibody against the cytosolic p65 subunit of the transcription factor nuclear factor κB (NFκB) to show that intracellular proteins were not detected by this staining method (S2 Fig).

### Archazolid increases the presence of extracellular collagen by reducing the expression and activity of cathepsin B

**Fig 8:**
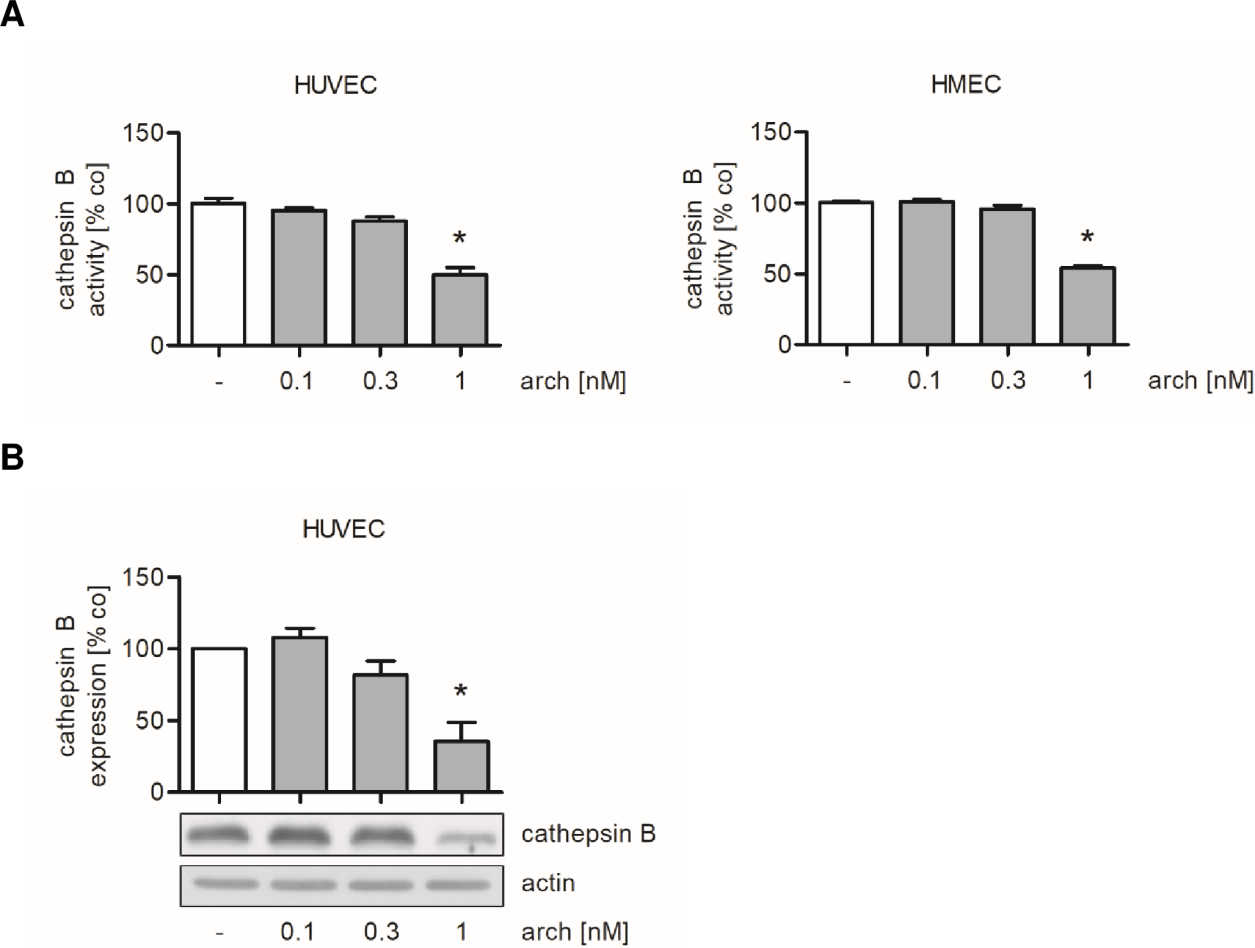
Archazolid decreases the activity and expression of cathepsin B in endothelial cells. Confluent HUVECs or HMEC-1 were treated with archazolid (arch) or DMSO (co) for 24 h. (A) Cathepsin B (catB) activity was measured in cell lysates of treated endothelial cells by using a fluorogenic cathepsin B substrate. Data are expressed as mean ± SEM (HUVECs n = 5; HMEC-1 n = 4). *p ≤ 0.05 versus DMSO control. (B) Western blot analysis of mature cathepsin B was performed and quantified densitometrically. Actin served as loading control. Data are expressed as mean ± SEM (n = 4). *p ≤ 0.05 versus DMSO control. One representative blot out of three independently performed experiments is shown.

It was reported that v-ATPase inhibition by archazolid impairs the activity of cathepsin B [28,36], a lysosomal enzyme that degrades extracellular matrix components including collagen [37–41]. As collagen is degraded by cathepsin B and the activation of cathepsin B depends on v-ATPase activity [28,36–38,42], we suggested that an accumulation of collagen on the surface of endothelial cells might be a consequence of an impaired functionality of cathepsin B. Therefore, an enzyme activity assay based on the proteolysis of a fluorogenic cathepsin B substrate was performed. In archazolid-treated HUVECs and HMEC-1 the activity of cathepsin B was strongly decreased by approximately 50% compared to control cells at an archazolid concentration of 1 nM (Fig 8A). In line with this result, western blot analysis showed that archazolid (1 nM) reduces the protein expression of the mature, active form of cathepsin B to less than 40% of the control in HUVECs (Fig 8B).

**Fig 9:**
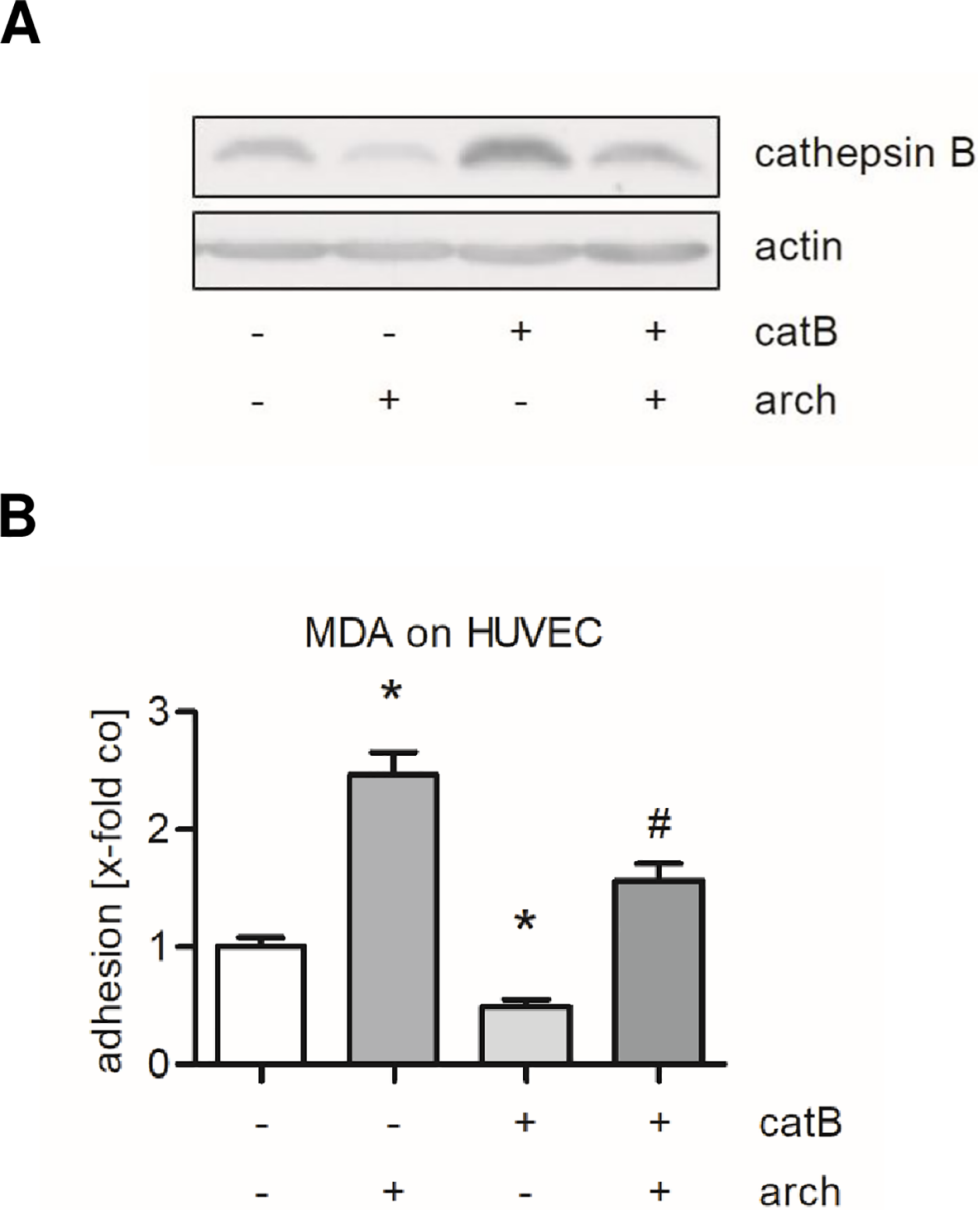
Overexpression of cathepsin B in endothelial cells attenuates both the basal and the archazolid-induced tumor cell adhesion. HUVECs were transfected with a plasmid containing human cathepsin B (catB) or the empty vector (pcDNA3.1(-)delta MCS). After 48 h cells were treated with 1 nM archazolid (arch) or DMSO (co) for 24 h. (A) The expression of cathepsin B was determined by western blot analysis. One representative blot out of three independently performed experiments is shown. Actin served as loading control. (B) Untreated MDA-MB-231 cells were labeled with CellTracker Green CMFDA Dye, added to the HUVEC monolayer and were allowed to adhere for 10 min. Non-adherent MDA-MB-231 cells were washed off. The adhesion was quantified by measuring the fluorescence signal using a plate reader. Data are expressed as mean ± SEM (n = 7). *p ≤ 0.05 versus DMSO (control transfection). ^#^p ≤ 0.05 versus 1 nM archazolid (control transfection).

To proof whether the archazolid-induced tumor cell adhesion is a consequence of the decreased amount of cathepsin B, HUVECs were transfected with a plasmid coding for human cathepsin B or with the empty vector as control. After 48 h, the transfected cells were treated with 1 nM archazolid. The level of cathepsin B after transfection and treatment was assessed by western blot analysis (Fig 9A). Overexpression of cathepsin B strongly diminished both the basal and the archazolid-induced adhesion of MDA-MB-231 cells (Fig 9B).

## Discussion

Targeting the proton pump v-ATPase for cancer therapy has gained great interest since its inhibition was reported to reduce the invasiveness of cancer cells and, most importantly, also metastasis [8,9]. Thus, intensive research related to v-ATPases was done in cancer cells, whereas there are only few studies investigating v-ATPases in endothelial cells indicating a role in migration, proliferation and possibly angiogenesis [22–24]. In the present study we used the myxobacterial natural product archazolid to investigate the consequences of v-ATPase inhibition in the endothelium on tumor-endothelial cell interactions.

For the first time, we were able to show a link between v-ATPase and the adhesion and transmigration properties of the endothelium. Inhibition of the v-ATPase in endothelial cells by archazolid significantly increased the adhesion of metastatic cancer cells and decreased the transendothelial migration of cancer cells which was attributed to augmented collagen levels on the surface on archazolid-treated endothelial cells. Of note, adhesion of the non-metastatic Jurkat cell line onto archazolid-treated endothelial cells remained unaffected. The archazolid-induced adhesion of tumor cells was independent from the endothelial cell adhesion molecules ICAM-1, VCAM-1, E-selectin and N-cadherin, as their expression was not regulated by the compound. However, we found that the archazolid-induced tumor cell adhesion was mediated by β1-integrins expressed on MDA-MB-231 breast cancer and PC-3 prostate cancer cells as blocking of the integrin β1 subunit on these tumor cells reversed the pro-adhesive effect of archazolid. In adhesion experiments on plastic coated with extracellular matrix components, we could show that MDA-MB-231 and PC-3 cells clearly favored the interaction with collagen, whereas the adhesion of non-metastatic Jurkat cells was largely independent from extracellular matrix proteins (S1B Fig). The different adhesion properties of metastatic cancer cells and Jurkat cells might be a result of the distinct integrin expression pattern of each cell line. MDA-MB-231 and PC-3 cells express α2β1- and α3β1-integrins, which represent collagen receptors [43,44], while Jurkat cells express α4β1-integrins but lack α2β1-, α3β1-integrins [44]. α4β1-integrins are receptors for VCAM-1 and fibronectin [35] and it has been shown that Jurkat cells interact with human endothelial cells that express VCAM-1 after cytokine treatment or cells transfected with VCAM-1 [45]. Our results are in line with previous studies showing that α2β1- and α3β1-integrin expressing MDA-MB-231 and PC-3 cells were able to rapidly attach to collagen in the cortical bone matrix. In contrast, Jurkat cells were not able to adhere [44] and might preferentially interact with cell adhesion molecules rather than with ECM proteins. α2β1- and α3β1-integrins can additionally act as laminin receptors [46] and at least α3β1-integrins recognize fibronectin [46,47]. Though expressing receptors for fibronectin and laminin, MDA-MB-231 and PC-3 cells adhered to fibronectin to a much lesser extent and did not adhere to laminin, probably due to lower affinities to these extracellular matrix components.

Importantly, v-ATPase inhibition by archazolid increased the surface levels of the extracellular matrix component collagen, which might explain that the increase of MDA-MB-231 and PC-3 cells onto archazolid-treated HUVECs is independent of endothelial cell adhesion molecules. By performing a live cell proteolysis assay, Cavallo-Medved *et al*. demonstrated ECM degradation, in particular of gelatin and collagen IV, in association with active cathepsin B in caveolae of endothelial cells during tube formation [40]. In addition, recent studies reported that v-ATPase inhibition impairs the activity of cathepsin B in cancer cells [28,36]. Therefore, we suggested that the accumulation of collagen on the endothelial surface might be a consequence of impaired cathepsin B activity or expression in endothelial cells. In fact, we confirmed the impairment of cathepsin B activity by archazolid as the expression levels of the mature active form of this enzyme was strongly reduced. Cathepsin B is synthesized as preprocathepsin B on membrane-bound ribosomes. Following transport to the Golgi apparatus, the preprocathepsin B is glycosylated with mannose-containing oligosaccharides. The targeting of procathepsin B to lysosomes is mannose-6-phosphate receptor-dependent and its dissociation from the receptor as well as its proteolytic processing into mature cathepsin B requires acidification of the compartment [48]. In cancer cells v-ATPase inhibition by archazolid impaired the mannose-6-phosphate receptor-mediated trafficking from the trans-Golgi network to prelysosomal compartments resulting in a decrease of active lysosomal proteases like cathepsin B [28]. We assumed that the archazolid-induced decrease in cathepsin B activity and expression was based on the same mechanism. Interestingly, overexpression of cathepsin B attenuated the archazolid-induced adhesion of breast cancer cells onto endothelial cells, indicating that the adhesion negatively correlates with the expression of cathepsin B.

As cathepsin B can also degrade other extracellular matrix components such as fibronectin and laminin [38,49], v-ATPase inhibition could lead to an accumulation of these proteins and an increased adhesion of cells expressing fibronectin or laminin receptors. However, we did not focus on these ECM components since they were not relevant for the adhesion of MDA-MB-231 and PC-3 cells. These cells predominantly adhered to collagen, while the adhesion of Jurkat cells is mostly independent from the ECM proteins collagen, fibronectin or laminin (S1B Fig).

Interestingly, the archazolid-induced MDA-MB-231 cell adhesion onto the endothelium resulted in a decrease in transendothelial migration of these cells. In contrast, Haidari *et al*. showed that β1-integrin-mediated adhesion of MDA-MB-231 and PC-3 cells onto HUVECs induces Tyr phosphorylation of vascular endothelial (VE)-cadherin, dissociation of catenin from the VE-cadherin complex and retraction of endothelial cells which facilitates transendothelial migration of tumor cells. Moreover, this Tyr phosphorylation of VE-cadherin is mediated by activation of the H-Ras/Raf/MEK/ERK signaling cascade. Inhibition of H-Ras in HUVECs blocked the transendothelial migration of MDA-MB-231 cells [50]. In hepatic cancer cells, archazolid reduces Ras/Raf/MEK/ERK signaling by altering the membrane composition and fluidity [51]. We assume that archazolid affects endothelial cells in a similar way leading to inhibition of Ras signaling and, therefore, reduced transendothelial migration of MDA-MB-231 cells.

Taken together, our study shows that archazolid reduces the activity and expression of cathepsin B in endothelial cells. As a result, the amount of collagen on the surface of endothelial cells was significantly upregulated, which finally resulted in an increased adhesion of the β1-integrin-expressing metastatic cancer cell lines MDA-MB-231 and PC-3 onto archazolid-treated endothelial cells, whereas the adhesion of non-metastatic Jurkat cells was unaffected. This study shows that the v-ATPase plays an important role in regulating the adhesion of cells expressing receptors for extracellular matrix components. Archazolid represents a promising tool to elucidate the role of v-ATPase in endothelial cells. Moreover, we for the first time linked the function of v-ATPase to the adhesion and transmigration of tumor cells onto endothelial cells as well as to the remodeling of the extracellular matrix on the surface of endothelial cells. The fact that the adhesion of metastatic tumor cells onto endothelial cells is increased while their transendothelial migration is reduced upon inhibition of endothelial v-ATPase by archazolid further supports the view of archazolid as a potential anti-metastatic compound.

## Supporting information

S1 Supporting Information (DOCX)

S1 Fig Adhesion of Jurkat cells onto endothelial cells and ECM components. (A) Confluent HUVECs were treated with archazolid (arch) or DMSO (co) for 24 h. (B) 24-well plates were coated with 10 μg/ml collagen (col), fibronectin (fn) or laminin (ln) or were left uncoated (co). (A, B) Untreated Jurkat cells were stained with CellTracker Green CMFDA Dye and added to the HUVEC monolayer (A) or to ECM-coated or uncoated wells (B). After 60 min of incubation, non-adherent cells were washed off. Adhesion was quantified by measuring the fluorescence signal using a plate reader. Data are expressed as mean ± SEM (n = 5). *p ≤ 0.05 versus co.
(TIF)

S2 Fig Control stainings of the cytosolic NFκB subunit p65. Method 1: Staining with 1^st^ antibody for 30 min at 4°C, fixation with 4% formalin, no permeabilisation with triton. Method 2: Fixation with 4% formalin, no permeabilisation with triton. Method 3: Fixation with 4% formalin, permeabilisation with triton. Each staining was performed once. Green: p65; blue: nuclei (Hoechst 33342). Scale bar represents 50 μm.
(TIF)
