# Supplementary material for: The vacuolar-type ATPase inhibitor archazolid increases tumor cell adhesion to endothelial cells by accumulating extracellular collagen
Source: PLoS One. 2018 Sep 11;13(9):e0203053. doi: 10.1371/journal.pone.0203053 (PMC6133348; doi:10.1371/journal.pone.0203053)
Supplement: S1 Supporting Information — (DOCX) [file pone.0203053.s001.docx]

**Supplementary Information**

**Materials and Methods**

**Cell culture**

Jurkat cells (ACC-282) were obtained from the Leibniz Institute DSMZ-German Collection of Microorganisms and Cell Cultures and cultivated in Roswell Park Memorial Institute medium (HyClone RPMI 1640, GE Healthcare) supplemented with 10 % FCS, 100 U/ml penicillin and 100 µg/ml streptomycin at 37 °C and 5 % CO_2_.

**Cell adhesion assay**

HUVECs were seeded in collagen G-coated 24-well plates and grown to confluence for two days before treatment. The cells were incubated with indicated concentrations of archazolid for 24 h. Untreated Jurkat cells were labeled with CellTracker Green CMFDA Dye (1 million cells per ml in 5 µM in serum-free DMEM, 37 °C) for 30 min before 100,000 cells per well were added to HUVECs and were allowed to adhere for 1 h at 37 °C. Non-adherent Jurkat cells were washed off three times with PBS containing Ca^2+^ and Mg^2+^. Tumor cell adhesion was determined by fluorescence measurement with an Infinite F200 pro microplate reader (Tecan) at 485 nm (excitation) and 535 nm (emission).

For the adhesion of Jurkat cells onto extracellular matrix (ECM) components 24-well plates were coated with collagen G (10 µg/ml in PBS), human plasma fibronectin (10 µg/ml PBS) or laminin-411 (10 µg/ml in DPBS containing Ca^2+^ and Mg^2+^) at 4 °C overnight. The adhesion of Jurkat cells onto these ECM components was carried out as described above (1 h adhesion at 37 °C).

**Immunofluorescence staining of viable and fixed cells**

Using the standard procedure of immunofluorescence stainings intra- and extracellular proteins are detected. To stain surface collagen on HUVECs, cells were incubated with a primary antibody on ice before fixation to ensure that surface proteins or antibodies are not endocytosed. To demonstrate that this method does not detect any intracellular proteins, cells were incubated with an antibody against the intracellular NFκB subunit p65 (rabbit, 1:400, sc-372, Santa Cruz Biotechnology, Heidelberg, Germany) on ice for 30 min. The cells were washed once with PBS containing Ca^2+^ and Mg^2+^ before they were fixed with Roti-Histofix (method 1 corresponding to the method used for detecting surface collagen). To show that the procedure of fixation makes the cells leaky and allows the entrance of antibody into the cells, fixed cells were directly incubated with the anti-p65 antibody for 2 hours at room temperature without permeabilization (method 2). Method 3 corresponds to the standard procedure of immunofluorescence stainings, in which cells were fixed, permeabilized with 0.2 % Triton X-100 in PBS (2 min, Sigma-Aldrich) and then incubated with the anti-p65 antibody for 2 h at room temperature. Alexa Fluor 488-conjugated anti-rabbit antibody (goat, 1:400, A11008, Life Technologies) was used as secondary antibody and Hoechst 33342 (1 µg/ml, Sigma-Aldrich) was used to visualize nuclei for all three methods.
