# Supplementary figures and images for: The vacuolar-type ATPase inhibitor archazolid increases tumor cell adhesion to endothelial cells by accumulating extracellular collagen
Source: PLoS One. 2018 Sep 11;13(9):e0203053. doi: 10.1371/journal.pone.0203053 (PMC6133348; doi:10.1371/journal.pone.0203053)

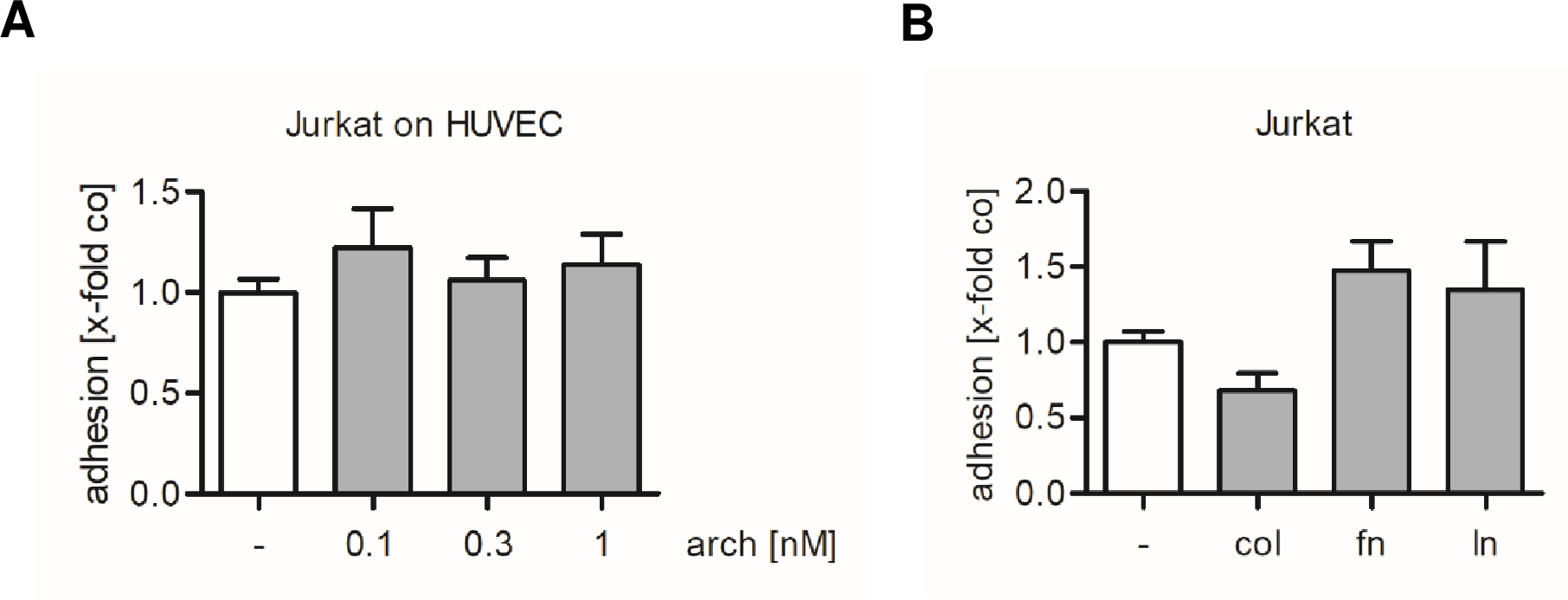

Supplement: S1 Fig — (A) Confluent HUVECs were treated with archazolid (arch) or DMSO (co) for 24 h. (B) 24-well plates were coated with 10 μg/ml collagen (col), fibronectin (fn) or laminin (ln) or were left uncoated (co). (A, B) Untreated Jurkat cells were stained with CellTracker Green CMFDA Dye and added to the HUVEC monolayer (A) or to ECM-coated or uncoated wells (B). After 60 min of incubation, non-adherent cells were washed off. Adhesion was quantified by measuring the fluorescence signal using a plate reader. Data are expressed as mean ± SEM (n = 5). *p ≤ 0.05 versus co. (TIF) [file pone.0203053.s002.tif]

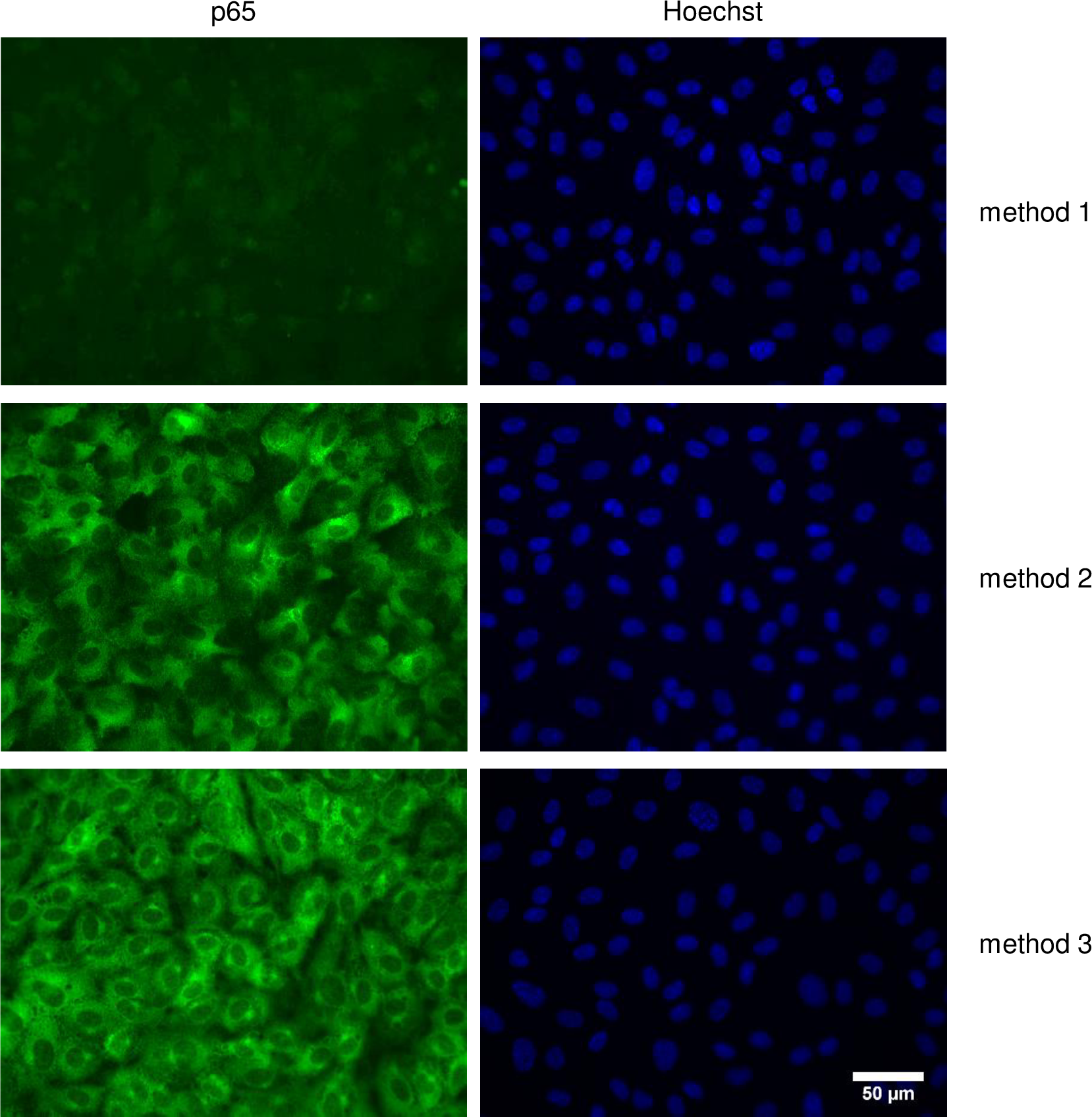

Supplement: S2 Fig — Method 1: Staining with 1st antibody for 30 min at 4°C, fixation with 4% formalin, no permeabilisation with triton. Method 2: Fixation with 4% formalin, no permeabilisation with triton. Method 3: Fixation with 4% formalin, permeabilisation with triton. Each staining was performed once. Green: p65; blue: nuclei (Hoechst 33342). Scale bar represents 50 μm. (TIF) [file pone.0203053.s003.tif]
